# Biological role and expression of translationally controlled tumor protein (TCTP) in tumorigenesis and development and its potential for targeted tumor therapy

**DOI:** 10.1186/s12935-024-03355-9

**Published:** 2024-06-04

**Authors:** An-Bu Liu, Jia Liu, Sheng Wang, Lei Ma, Jun-Fei Zhang

**Affiliations:** 1 Department of Emergency Medical, General Hospital of Ningxia Medical University, Yinchuan, 750000 Ningxia China; 2 Medical Experimental Center, General Hospital of Ningxia Medical University, Yinchuan, 750000 Ningxia China; 3 School of Clinical Medicine, Ningxia Medical University, Yinchuan, 750000 Ningxia China

**Keywords:** Translationally controlled tumor protein (TCTP), Tumorigenesis, Cell proliferation, Cell death, Cell migration

## Abstract

Translationally controlled tumor protein (TCTP), also known as histamine-releasing factor (HRF) or fortilin, is a highly conserved protein found in various species. To date, multiple studies have demonstrated the crucial role of TCTP in a wide range of cellular pathophysiological processes, including cell proliferation and survival, cell cycle regulation, cell death, as well as cell migration and movement, all of which are major pathogenic mechanisms of tumorigenesis and development. This review aims to provide an in-depth analysis of the functional role of TCTP in tumor initiation and progression, with a particular focus on cell proliferation, cell death, and cell migration. It will highlight the expression and pathological implications of TCTP in various tumor types, summarizing the current prevailing therapeutic strategies that target TCTP.

## Introduction

Malignancy is one of the most critical public health issues globally. Previous research data has demonstrated that cancer presents the greatest clinical, social, and economic burden among all human diseases [1]. According to statistics, the total number of newly diagnosed cancer cases in 2018 was estimated to be around 18 million. The three most common types of cancer were lung cancer (2.09 million cases), breast cancer (2.09 million cases), and prostate cancer (1.28 million cases) [2]. Furthermore, the report has indicated that cancer is the second leading cause of mortality, surpassed only by cardiovascular diseases, resulting in 8.97 million deaths [3]. More specifically, the three leading causes of mortality in the general population attributed to cancer can be, in descending order, respiratory cancer, hepatocellular carcinoma (HCC), and gastric cancer [2]. The aforementioned data underscores the critical necessity of researching tumor mechanisms and therapeutic targets as key requirements for seeking more effective methods of cancer management. This pursuit will contribute to lowering the mortality rate of cancer patients while concurrently improving their quality of life throughout the course of therapy. Targeted therapy is a therapeutic approach that targets the cancer-triggering sites identified at the molecular level. Specifically, drugs entering the body may selectively target the cancer sites to combine and kill tumor cells without affecting the adjacent healthy tissue. Currently, commonly considered targets for tumor therapy include CD20, HER2, VEGF, EGFR, KIT, etc. [4, 5]. Drugs designed for the above targets have already entered clinical application and participated in the management of carcinoma, such as imatinib, bevacizumab, and cetuximab [5–7]. However, with the prolongation of the therapeutic process, patients receiving targeted therapy develop drug resistance, which seriously affects the clinical outcome and prognosis of tumor patients. Therefore, it is particularly important to find new tumor therapeutic targets.

Translationally controlled tumor protein (TCTP) is a protein that exhibits high conservation across multiple species [8]. One of its key features is that TCTP mRNA has the ability to translate and control the complete sequence and structure of other mRNAs [9, 10]. TCTP has an extensive evolutionary history, and its mRNA is broadly expressed in diverse eukaryotic organisms, including yeast, animals, and plants. Nonetheless, its expression level varies across various cell types and developmental stages [11–13]. Previous studies have indicated that the cDNA of human TCTP encodes a protein with a calculated molecular weight of 19 kDa (172 amino acids) [14]. Sequence analysis has revealed that TCTP is highly conserved and lacks homology with other proteins. The broad expression of TCTP in mammalian tissues highlights its significance in normal physiological processes. To date, numerous studies have demonstrated the crucial role of TCTP in a wide range of cellular pathophysiological processes, including cell cycle regulation, apoptosis, heat shock response (HSP), gene expression control, stress response, immune response, and tumorigenesis [8, 15–25]. Moreover, the extracellular function of TCTP, specifically its ability to release histamine, has been identified in animal immune research [26]. TCTP is overexpressed in a variety of tumors, such as breast cancer, colorectal cancer, prostate cancers, glioblastoma, and melanoma, etc. [27–31]. Furthermore, several studies have indicated that TCTP is implicated in the initiation and progression of various types of tumors [26, 32, 33]. However, the existing research on TCTP and its involvement in tumor development is relatively fragmented, calling for a comprehensive review and synthesis to pave the way for future in-depth investigations. Therefore, the primary objective of this review is to provide an in-depth analysis of the functional role of TCTP in tumorigenesis and development, with a particular focus on cell proliferation, cell death, and cell migration. Additionally, we will highlight the expression and pathological implications of TCTP in various tumor types and briefly summarize the current prevailing therapeutic strategies that target TCTP.

## Biological role of TCTP in tumorigenesis and development

Current research suggests that TCTP is involved in the initiation and development of tumors by promoting cell proliferation, inhibiting cell death, and enhancing cell migration. Therefore, we may proceed to elaborate on the above three aspects.

### TCTP facilitates cell proliferation in cancer

A study conducted on Drosophila has demonstrated that knocking down TCTP expression in Drosophila leads to a decrease in cell number, volume, and organ size, ultimately resulting in the mortality of Drosophila larvae [32]. Moreover, several studies have corroborated that TCTP exhibits elevated expression levels in cells with high proliferative activity [11, 34]. Hence, it is plausible to suggest that TCTP plays a pivotal role in cell proliferation. Present investigations into TCTP’s function in cell proliferation predominantly center on its modulation of the mammalian target of rapamycin (mTOR) signaling pathway and cell cycle regulation.

#### mTOR signaling pathway

mTOR, short for the mammalian target of rapamycin, is encoded by the FRAP1 gene. It belongs to the phosphatidylinositol 3-kinase-related kinase (PIKK) protein family and is considered an atypical serine/threonine protein kinase. mTOR plays a pivotal role in cellular growth, apoptosis, autophagy, and metabolism. mTOR functions within cells primarily through two distinct complexes, known as mTORC1 and mTORC2. Rheb, a small GTPase, acts as a strong activator of mTORC1 kinase activity when bound to GTP (guanosine triphosphate). Rheb is subject to negative regulation by the tuberous sclerosis complex (TSC). When TSC is active, it boosts the conversion of Rheb-GTP to Rheb-GDP, thereby suppressing Rheb-mediated activation of the entire mTORC1 cascade. The signaling pathways of mTORC1 primarily involve positive regulation by the PI3K/AKT pathway and negative regulation by the adenosine monophosphate-activated protein kinase (AMPK) pathway [35–38]. Additionally, the activated receptor Ras signaling pathway can activate ERK and inhibit TSC, ultimately resulting in the activation of mTORC1 [39, 40].

**Fig. 1:**
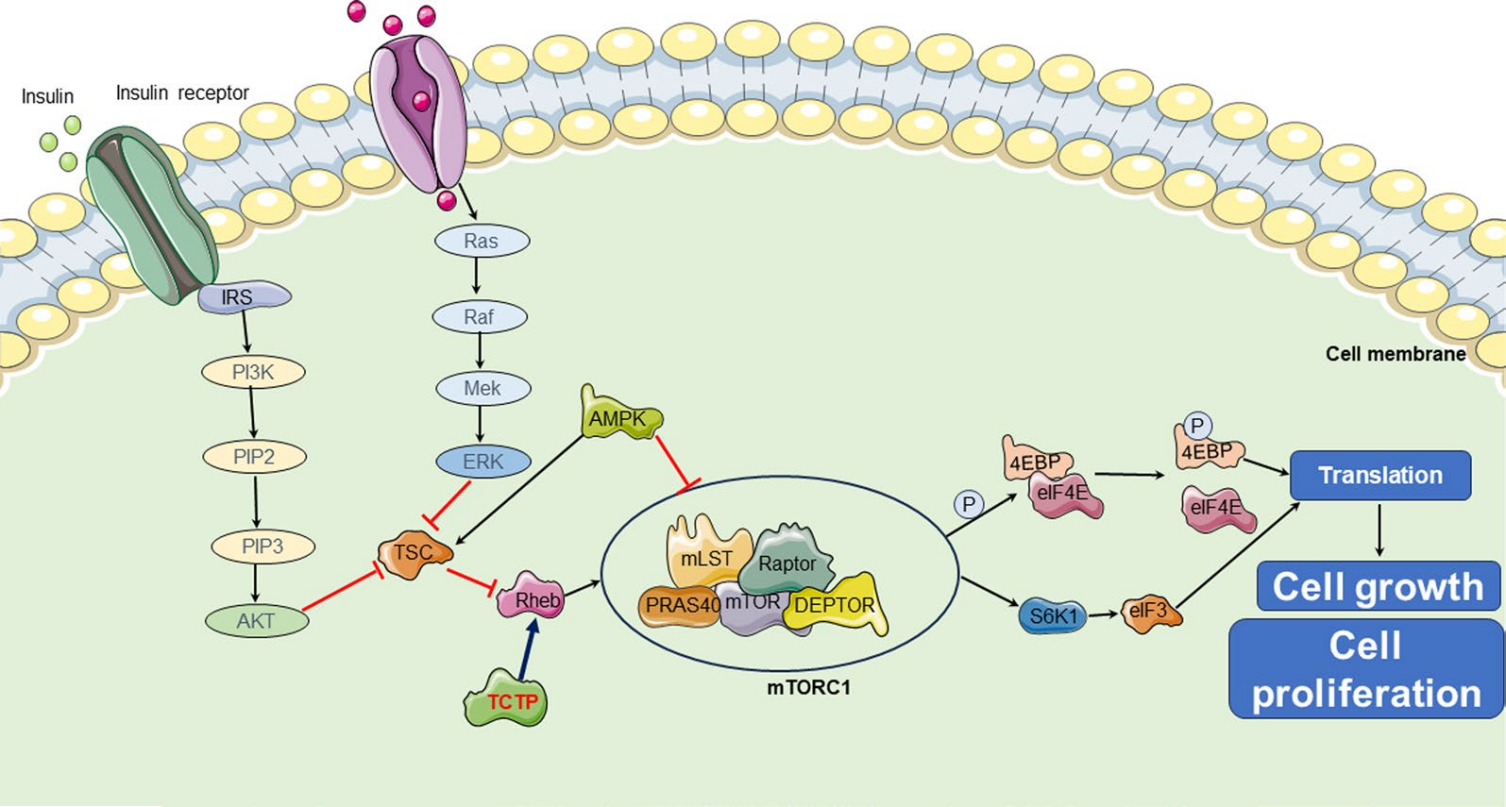
TCTP regulate mTOR signaling pathway to participate tumor initiation. TCTP can promote Rheb activity and activate mTORC1. activated mTORC1 can facilitate translation and ultimately trigger cell growth and cell proliferation by directly activating S6K1 or phosphorylating 4EBP1, thereby dissociating from eIF4E and allowing eIF4E. *mTOR* mammalian target of rapamycin, *IGF-1* insulin-like growth factor 1, *PI3K* phosphoinositide 3-kinase, *PIP2* phosphatidylinositol 4,5-bisphosphate, *PIP3* phosphatidylinositol 3,4,5-trisphosphate, *AMPK* adenosine monophosphate-activated protein kinase, *S6K1* subunit 6 kinase 1, *TSC* tuberous sclerosis complex, *4E-BP1* 4E-binding protein 1, *eIF3* eukaryotic initiation factor 3, *eIF4E* eukaryotic initiation factor 4E

Research in Drosophila has indicated that TCTP can bind to Rheb and act as a guanine nucleotide exchange factor (GEF) for this molecule [32]. However, the mechanism of interaction between TCTP and Rheb is still debated by some researchers [41, 42]. Le et al. found that the 14-3-3 protein might be necessary for TCTP to bind with Rheb, which could provide a possible explanation for why the state of the 14-3-3 protein may affect the reproducibility of the interaction between TCTP and Rheb [43]. Despite the controversy surrounding the mechanism of interaction between TCTP and Rheb, the experimental results mentioned above may support the hypothesis that "TCTP is likely to facilitate cell proliferation and growth through the mTOR-mediated pathway". This hypothesis has been subsequently validated in further research [44]. In summary, an increase in the expression or activity of TCTP can promote Rheb activity and activate mTORC1 (Fig. 1).

Activated mTORC1 primarily promotes protein synthesis by phosphorylating two key downstream effectors, ribosomal protein subunit 6 kinase 1 (S6K1) and eukaryotic translation initiation factor 4E-binding protein 1 (4E-BP1), thereby regulating cellular proliferation and growth. When nutrients are abundant, mTORC1 can directly phosphorylate S6K1, thereby activating several substrates that promote mRNA translation initiation, such as the eukaryotic initiation factor 3 (eIF3) complex [45, 46]. Another target of mTORC1 is 4EBP1, which can suppress translation by binding to eukaryotic initiation factor 4E (eIF4E), a cap-binding protein that recruits the 40S ribosomal subunit to the 5ʹ end of mRNA for translation initiation. The interaction between 4EBP1 and eIF4E leads to translational repression. Upon activation of mTORC1, 4EBP1 is phosphorylated, resulting in its dissociation from eIF4E and allowing eIF4E to initiate translation [47].

Based on the above, it is reasonable to speculate that TCTP enhances Rheb activity, which in turn activates the mTOR1 complex. This mechanism could potentially explain why TCTP plays a significant biological role in tumor cell proliferation by responding to various signaling stimuli. However, further research is warranted to delve into the underlying mechanisms. Additionally, the mechanism of interaction between TCTP and mTORC2 remains elusive and requires further investigation in the future.

#### Cell cycle

The cell cycle of eukaryotic organisms is partitioned into interphase and mitotic phase. Interphase is chiefly comprised of the G1 phase, S phase, and G2 phase, while the mitotic phase is also referred to as the M phase [48]. During the cell cycle, a multitude of cellular events such as DNA replication, gene transcription, protein translation, and post-translational modifications, in a coordinated manner. Moreover, the cell cycle is intricately linked with cell proliferation and survival, and it is tightly regulated by multiple mechanisms [49]. Polo-like kinase 1 (PLK1) and checkpoint protein with forkhead-associated and ring finger domains (CHFR), which possess specific structural domains, play crucial roles in the regulation of the cell cycle. CHFR protein is classified as an E3 ubiquitin ligase, playing a regulatory role in the cell cycle. Normally, CHFR exists in an inactive form during physiological conditions and is incapable of undergoing ubiquitination. However, upon microtubule damage, CHFR becomes activated and facilitates the ubiquitination of PLK1, resulting in the degradation of PLK1. This process ultimately triggers a delay in the transition from the G2 phase to the M phase [50]. PLK1 is involved in the regulation of entry into mitosis and the G2/M checkpoint [51, 52]. Moreover, it has diverse functions in spindle assembly and disassembly processes, mainly involving the coordination of the centrosome and cell cycle, as well as the regulation of spindle assembly and chromosome separation [53, 54]. Additionally, PLK1 can regulate cytokinesis in late mitosis by phosphorylating the mitotic kinase-like protein l, which can control cytoplasmic separation and membrane formation [54–56]. Hence, loss of PLK1 function has the potential to trigger cell cycle arrest and, subsequently, apoptosis. Conversely, overexpression of PLK1 is frequently linked to abnormalities in centrosomes, improper chromosome segregation, and tumor formation.

While the mTOR pathway may indirectly regulate the cell cycle through processes such as protein synthesis and energy metabolism, TCTP may appear to have a more direct and pivotal influence on cell cycle regulation. Studies have shown that TCTP is highly expressed in actively dividing cells, which has sparked interest in exploring the mechanism by which TCTP controls the cell cycle and participates in cellular proliferation [11, 15, 57, 58]. Furthermore, TCTP can interact with microtubules as a microtubule-binding protein and exhibit the ability to bind microtubules during the G1, S, G2, and M phases of the cell cycle. It was associated with the midzone spindle but dissociated from the spindle after the midzone stage [59]. Two primary mechanisms have been identified for the regulation of the cell cycle by TCTP. Firstly, TCTP can interact with CHFR, thereby enabling its interaction with microtubules and ultimately playing a role in cell cycle regulation. Research has demonstrated that under stress-induced microtubule depolymerization, the interaction between TCTP and CHFR weakens, resulting in aberrant cell cycle progression [18]. Alternatively, TCTP can be phosphorylated by PLK1, potentially reducing its affinity for microtubules or CHFR [60]. Yarm et al. observed that blocking the phosphorylation site of PLK1 on TCTP led to a substantial increase in the number of multinucleated cells, indicating the inhibition of normal mitosis [61]. These findings underscored the critical role of TCTP in cell cycle regulation. Proper phosphorylation of TCTP by PLK1 appeared to be essential for precise completion of mitosis and normal cellular physiological function [62].

Multiple studies have indicated that dysregulation or mutation of TCTP expression levels in mammalian cells can result in cell cycle arrest, alterations in microtubule stability and cell morphology, eventually contributing to tumorigenesis [63]. Neuroblastoma patients with adverse clinical and pathological characteristics had higher expression levels of both PLK1 and phosphorylated TCTP, and furthermore, there was a positive correlation between their expression [64]. Moreover, increased levels of both PLK1 and TCTP are indicative of poorer prognosis. Jeong M et al. also demonstrated that targeting the mTORC1 signaling pathway, as well as S6K, AKT, and PLK1, can promote the degradation of TCTP, thereby enhancing the sensitivity of lung cancer cells to DNA-damaging drugs [65]. In recent years, modulating cell cycle and proliferation by targeting TCTP has emerged as a novel approach in tumor therapy. Lv et al. discovered that the suppression of protein phosphatase 2A using a small molecule LB100 can enhance the phosphorylation of PLK1, TCTP, and Cdk1 within cells, while decreasing p53 levels. This mechanism was involved in cell cycle arrest, mitotic catastrophe, DNA damage repair blockade, and suppression of cell proliferation, consequently increasing the sensitivity of nasopharyngeal carcinoma to radiotherapy [66].

In summary, TCTP can regulate cell growth and protein synthesis by modulating mTOR signaling, and influence the cell cycle through PLK1, CHFR, and microtubule activity. These mechanisms collectively contributed to the promotion of cell proliferation and neoplasm progression. Consequently, targeting TCTP holds promise as a potential strategy for malignant tumor therapy in the future.

### TCTP inhibits cell death in cancer

In recent years, multiple studies have indicated that TCTP may affect tumor occurrence and progression by influencing cell death processes, particularly apoptosis and autophagy. In this review, we aim to elaborate on the role of TCTP in cell death from these two perspectives.

#### Apoptosis

**Fig. 2:**
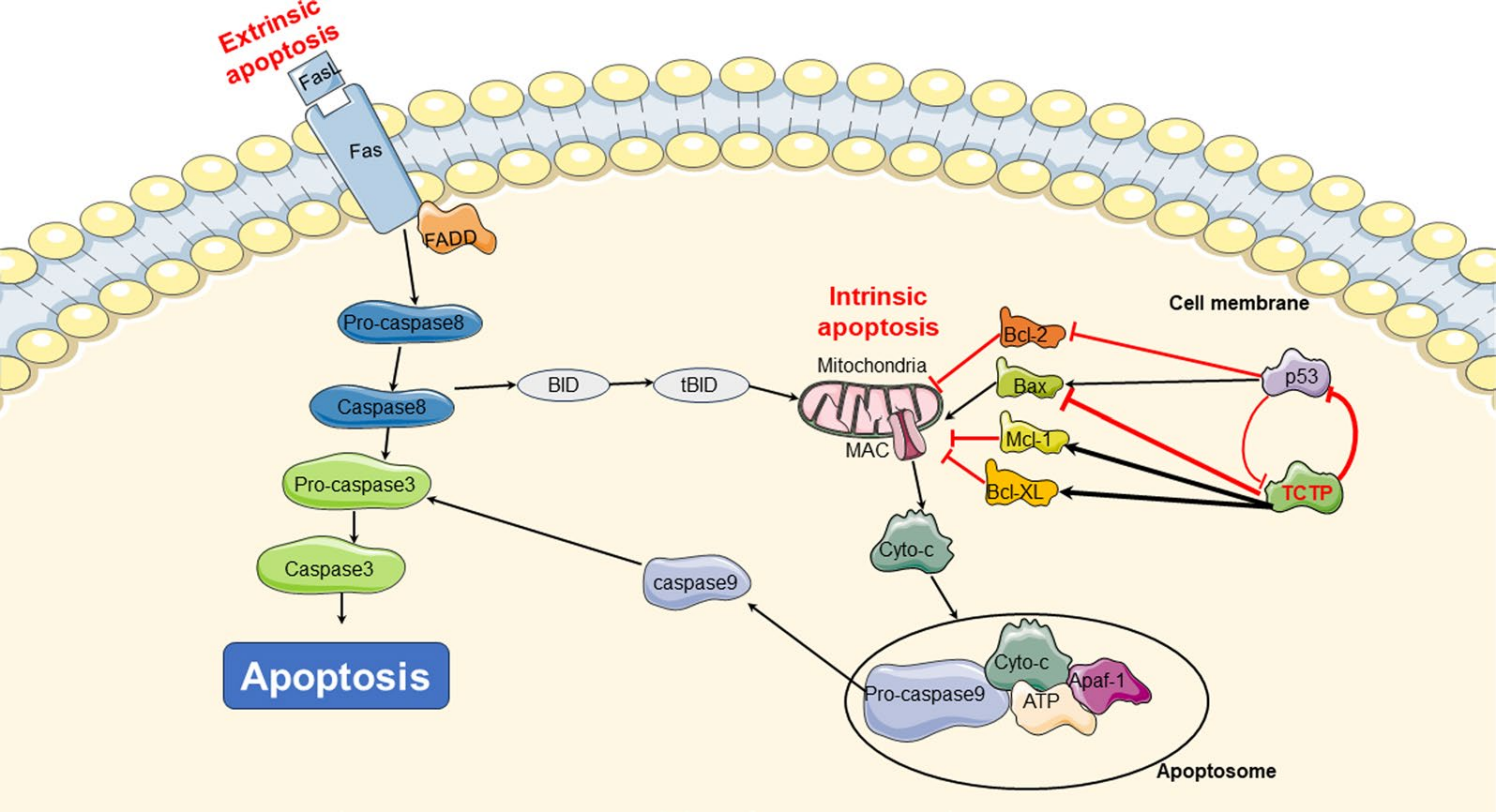
TCTP inhibit cell apoptosis to facilitate tumor initiation. TCTP can suppress cell apoptosis by stabilizing anti-apoptotic Bcl-2 family proteins, Mcl-1 and Bcl-xL and by inhibiting activation of pro-apoptotic Bcl-2 family protein, Bax. TCTP inhibits p53-dependent apoptosis by downregulating the protein. Additionally, there is a negative feedback loop exists between p53 and TCTP. *cyto-C* cytochrome c, *SMAC* second mitochondria-derived activator of caspase, *Apaf-1* apoptotic protease activating factor-1

Apoptosis refers to the programmed death of cells that is controlled by genes to maintain internal environment stability. Its main characteristics include chromatin condensation, DNA fragmentation, appearance of apoptotic bodies, and externalization of phosphatidylserine [67]. Tumor development primarily involves two pathways of cell apoptosis, including extrinsic and intrinsic apoptotic pathway. Dysregulation of cell apoptosis can be a common mechanism underlying tumorigenesis, and the modulation of cell apoptosis has been utilized in targeted therapy for malignant carcinoma. Recent studies have provided evidence that TCTP may contribute to tumorigenesis by inhibiting apoptosis, particularly the intrinsic apoptotic pathway [68–70]. For example, in HeLa cells, overexpression of TCTP has been shown to block cell death induced by cytotoxic drugs through the inhibition of intrinsic apoptosis [71]. Upon exposure to various stressors, biochemical substances, and cytokines may be secreted abnormally, resulting in mitochondrial dysfunction. This leads to the release of cytochrome c from the mitochondria and an increase in the expression of the pro-apoptotic protein Bax. Simultaneously, an apoptosome is formed consisting of APAF1, cytochrome c, ATP, and caspase-9. This apoptosome leads to activation of caspase-9. The activated caspase-9 then initiates cell apoptosis through proteolytic cleavage, subsequently activating the executioner caspase (3 and 7), which play crucial roles in executing the apoptotic process. Current researches have provided compelling evidence indicating that TCTP mainly exerts its anti-apoptotic effects by downregulating the expression or activity of pro-apoptotic proteins while upregulating the expression or activity of anti-apoptotic proteins. This leads to the suppression of apoptotic signaling pathways, ultimately facilitating tumor development [17, 70, 72, 73] (Fig. 2).

##### Bcl-2 family

The Bcl-2 family plays a pivotal role in the regulation of cellular apoptosis. This protein family encompasses pro-apoptotic members such as BH3-Only and BH1-3, as well as anti-apoptotic proteins like Bcl-2 and Bcl-w. These two categories of proteins work in concert during the process of cell apoptosis, jointly determining whether cells enter the apoptotic program by mediating intrinsic or mitochondrial apoptotic signaling pathways. In addition to their role in cellular energy production, mitochondria also serve as critical regulators of apoptosis by harboring various pro-apoptotic proteins. Mitochondrial outer membrane permeabilization (MOMP) regulates the permeability of the mitochondrial membrane to pro-apoptotic proteins, ultimately determining whether these proteins leak into the cytoplasm and contribute to pathological processes. Interestingly, members of the Bcl-2 protein family, such as Bax and BH3, can modulate the aforementioned MOMP process [74–77]. Upon activation, Bax undergoes dimerization on the mitochondrial membrane, which in turn boosts the formation of MOMP pores, thereby allowing pro-apoptotic proteins to enter the cell [78]. Conversely, proteins such as Bcl-2 and Mcl-1 restrain the formation of MOMP pores, preventing the leakage of pro-apoptotic proteins into the cytoplasm and subsequent apoptosis [79]. They exert their function by sequestering pro-apoptotic factors, such as Bax or Bak, thereby preventing MOMP [80]. TCTP can enhance the function of pivotal proteins, including Bcl-xL and Mcl-1, in the mitochondrial apoptotic pathway [81–84].

The N-terminal of TCTP specifically binds to the BH3 domain of Bcl-xL, which is responsible for heterodimerization and homodimerization between members of the Bcl-2 protein family [85]. The specific binding of TCTP to Bcl-xL can effectively prevent Bax-dependent release of cytochrome c induced by Bcl-xL. Moreover, TCTP can inhibit the ubiquitination of Bcl-xL, thereby declining the turnover of Mcl-1 and ultimately suppressing Mcl-1-mediated cell apoptosis [86]. It is noteworthy that TCTP also disrupts the dimerization of the pro-apoptotic Bcl-2 family protein Bax, thereby exerting an inhibitory effect on cell apoptosis [17]. To investigate the interaction between TCTP and Bax, L. Susini et al. refined the crystal structure of human TCTP and discovered a structural similarity between the H2-H3 helix of TCTP and the H5-H6 helix of Bax. Furthermore, using site-directed mutagenesis technology, TCTP and Bax were found to participate in the regulation of mitochondrial membrane permeability during apoptosis. Subsequently, it was demonstrated that TCTP could counteract cell apoptosis by inserting itself into the mitochondrial membrane and inhibiting Bax dimerization. This effectively prevented the formation of the MAC pore and inhibited the entry of pro-apoptotic factors into the cytoplasm [17].

Additionally, TCTP can exert its anti-apoptotic effect by enhancing the stability of Mcl-1 [82, 83]. It has been observed that TCTP specifically interacts with Mcl-1 through a mechanism that is currently not fully understood [83]. It has been reported that in a mouse macrophage cell line infected with Leishmania parasites, TCTP can stabilize Mcl-1 and prevent its ubiquitination, thereby inhibiting proteasome-mediated degradation of Mcl-1 [83]. Indeed, it has been experimentally established that Mcl-1 is a target of the E3 ubiquitin ligase Mule, and their interaction is regulated by Bcl-2 family proteins that possess BH3 domains [80, 87]. Structural analysis has revealed that Mule harbors a BH3 domain capable of binding to the BH3 binding groove of Mcl-1, suggesting a potential direct competition between TCTP and Mule on the same surface of Mcl-1, ultimately influencing the fate of Mcl-1 and its stability [87]. Additionally, Zhang et al. have demonstrated that Mcl-1 can act as a partner of TCTP, thereby contributing to the maintenance of cellular TCTP levels [83]. These results emphasize the multifaceted interactions of TCTP with the Bcl-2 family, which collectively regulate apoptotic processes in cells.

##### p53 protein

The p53 protein is the product of the p53 gene and acts as a transcription factor to promote the expression of a series of pro-apoptotic genes. Previous studies have demonstrated that p53 mutations are detected in approximately 50% of all cancer cases, and the impairment of this protein's function serves as a prominent factor in cancer initiation [88]. During tumorigenesis, p53 induces cell apoptosis by upregulating the transcriptional expression of pro-apoptotic proteins such as Bax and Bak, while inhibiting the activity of the anti-apoptotic protein Bcl-2 [89, 90]. It is well established that a negative feedback loop exists between p53 and TCTP, whereby their interaction regulates various cellular processes [73]. Specifically, TCTP can promote the degradation of p53 by competing with NUMB for binding to p53-MDM2-containing complexes. This competition leads to the inhibition of mouse double minute 2 homolog (MDM2) auto-ubiquitination and, subsequently, the promotion of MDM2-mediated ubiquitination and degradation of p53, thereby inhibiting apoptosis and promoting cancer development. Silencing TCTP leads to an increase in p53 expression, facilitating tumor regression. Furthermore, p53 can inhibit TCTP expression, initiating a cascade of events including the inhibition of cell proliferation, arrest of the cell cycle, and an elevation in the rate of apoptosis during tumorigenesis.

Researchers have performed immunohistochemical (IHC) and ultrastructural analyses on HIO180 non-transformed ovarian epithelial cells, as well as OVCAR3 and SKOV3 ovarian epithelial cancer cells expressing low levels of inducible p53 [91]. Their results demonstrated a significant negative correlation between TCTP levels and p53 expression in these ovarian cancer cell lines [91]. Beyond its role in facilitating tumor development by downregulating p53 expression, TCTP overexpression can also enhance tumor migration and invasion. un et al. conducted a study on human lung cancer specimens and found that overexpression of TCTP led to a downregulation of E-cadherin and p53 expression, resulting in an increase in neoplasm migration and invasion [92]. It is believed that TCTP can inhibit p53 through various mechanisms, triggering the promotion of tumor development. Researchers performed transient transfection experiments and demonstrated that overexpression of TCTP can suppress p53-mediated apoptotic activity by boosting the degradation of p53 [31]. Furthermore, TCTP had the ability to bind to p53 and disrupt its stability in the A549 human lung cancer cell line, ultimately hindering cell apoptosis [22]. Given TCTP’s regulatory effect on p53, it is considered a promising therapeutic target for cancer treatment. Research has demonstrated that disrupting the binding of TCTP to p53 binding sites can increase p53 expression, which in turn facilitates a dose-dependent decrease in CDK2, CDK4, CDK6, cyclin D1, and cyclin D3. This ultimately triggers G0/G1 cell cycle arrest and exerts a therapeutic effect in cancer [93]. Furthermore, an in vivo xenograft study has confirmed the enhanced radiosensitivity of TCTP-downregulated A549 cells, thereby strengthening the therapeutic efficacy [94].

In addition to facilitating the function of Bcl-XL and Mcl-1 as well as suppressing the p53 protein, TCTP can also participate in the unfolded protein response (UPR) and contribute to the inhibition of apoptosis. One of the key players in the UPR is inositol-requiring enzyme 1α (IRE1α), which possesses protein kinase and endoribonuclease activities. Once the protein overload in the endoplasmic reticulum becomes overwhelming, IRE1α is ultimately responsible for inducing cellular apoptosis [95]. Pinkaew et al. discovered that TCTP can bind to phosphorylated IRE1α, subsequently restraining the activation of the JNK apoptotic pathway [96].

#### Autophagy

Autophagy is a cellular process that involves the transport of cellular proteins and organelles to lysosomes for degradation by lysosomal hydrolases, thereby playing a crucial role in maintaining metabolic homeostasis [97, 98]. The two most studied autophagy-related pathways are the mTORC signaling pathway, which exerts a negative regulatory role on autophagy, and the AMPK signaling pathway, which plays a positive regulatory role in cellular autophagy. Furthermore, mTORC can negatively regulate autophagy by competing with AMPK.

Autophagy plays a dual role in tumorigenesis, encompassing both the inhibition of tumor growth and the sustenance of cancer cell survival. Consequently, the regulation of autophagy has emerged as a prominent area of research in the field of tumor biology and targeted therapies. Initially, autophagy was thought to primarily inhibit tumor growth by suppressing malignant cell proliferation [99]. However, recent studies have shown that the mechanism for certain anti-cancer drugs is linked to the induction of autophagy in cancer cells [100, 101]. Recent studies have indicated that autophagy may serve as a mechanism of drug resistance to tumors [102–104]. Specifically, autophagy in traditional cancer treatments provides cells with an alternative survival route that circumvents cell death, potentially leading to treatment resistance. Therefore, studying autophagy within the framework of cancer therapy and investigating the feasibility of combining autophagy inhibitors with standard cancer treatments represents a promising approach to overcoming treatment resistance.

**Fig. 3:**
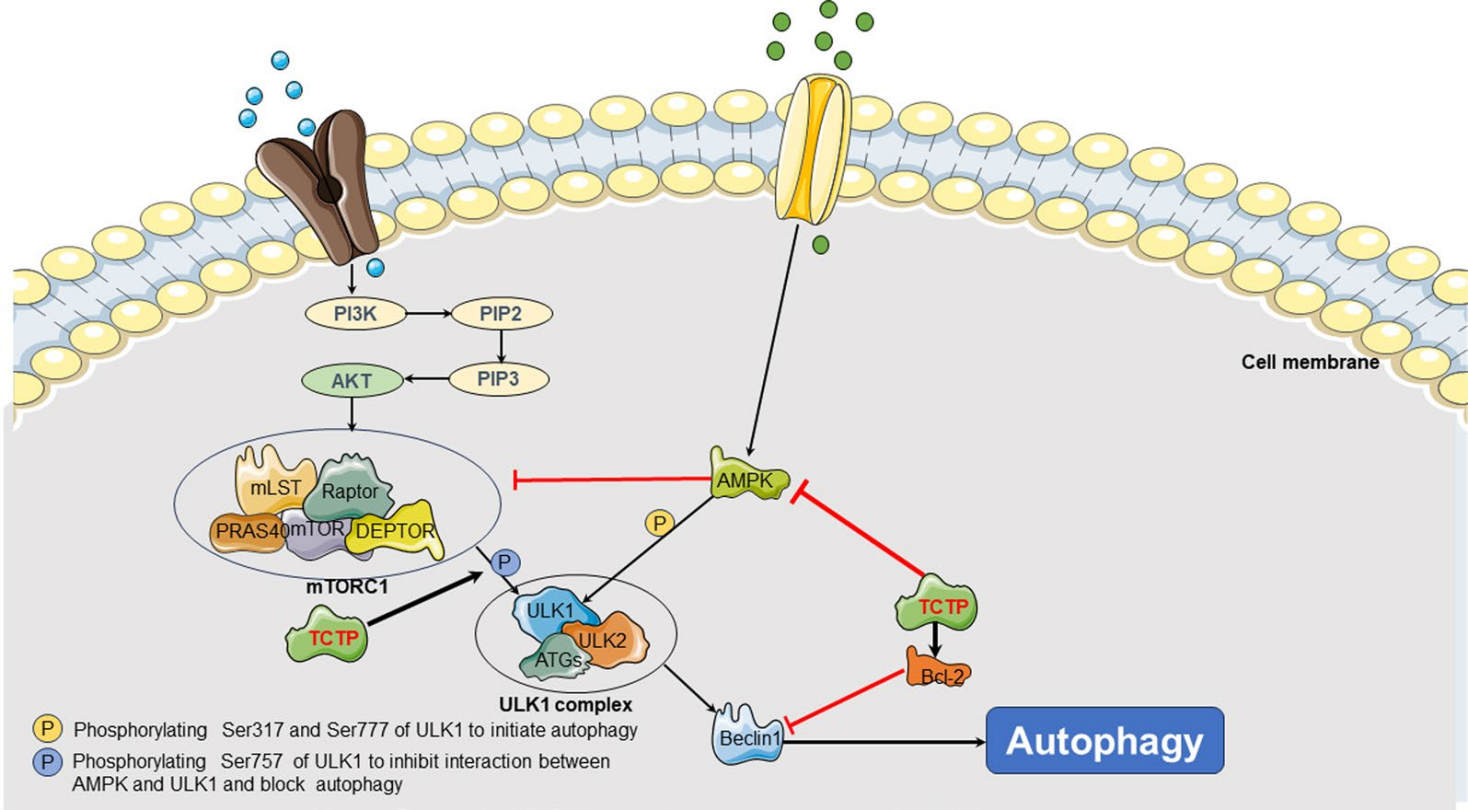
TCTP can promote tumorigenesis by regulate autophagy. TCTP can inhibit autophagy by suppressing the AMPK signaling pathway and promoting the mTORC signaling pathway. Additionally, TCTP can modulate autophagy through its effects on Beclin1 and Bcl-2

Currently, only a few studies have reported on the relationship between TCTP and autophagy. Early studies suggested that TCTP promoted autophagy [105]. Whereas, most subsequent research has indicated that TCTP may restrain cellular autophagy [106, 107]. A study has demonstrated that the downregulation of TCTP expression facilitates the formation and maturation of autophagosomes, as observed through the formation of LC3 puncta and co-localization of LC3 with the lysosomal marker LAMP1 [106]. Moreover, research conducted on HeLa cells has revealed that the downregulation of TCTP expression can enhance the phosphorylation of AMPK and its downstream signaling pathways, while inhibiting downstream effectors of the mTORC1 pathway, including p-EIF-4EBP1, p-RPS6KB, and p-ULK1 (Ser757) [107]. Ultimately, these alterations can lead to the promotion of cellular autophagy. Furthermore, the knockdown of TCTP has been demonstrated to synergistically suppress the mTORC1 pathway in combination with rapamycin [45] This finding indicated that TCTP may represent a promising target for overcoming rapamycin resistance in cancer therapy. In addition to suppressing autophagy by inhibiting the AMPK signaling pathway and promoting the mTORC signaling pathway, TCTP might also modulate autophagy through its effects on Beclin1 and Bcl-2. The Beclin1 gene, also referred to as the BECN1 gene, is a homolog of yeast ATG6 and is a specific gene involved in autophagy in mammals [108]. The expressed product of the Beclin1 gene functions as a subunit of the Class III PI3K complex, which interacts with autophagic precursors to initiate the formation of autophagosomes [109]. Bcl-2 can bind with Beclin-1 and disrupt the Beclin-1/PI3K complex, which may subsequently block autophagy [110, 111]. It is noteworthy that TCTP facilitates the expression of Bcl-2 while leaving the expression of Beclin-1 unaffected. This induces the inhibition of autophagosome formation mediated by Beclin-1 [106, 112] (Fig. 3).

Overall, TCTP can be implicated in tumorigenesis through its regulation of apoptosis and autophagy. However, further investigations are required to elucidate the precise mechanisms underlying the role of TCTP in regulating cell death, in order to provide additional theoretical support for targeted therapy in malignant carcinoma.

### TCTP promotes cell migration in cancer

**Fig. 4:**
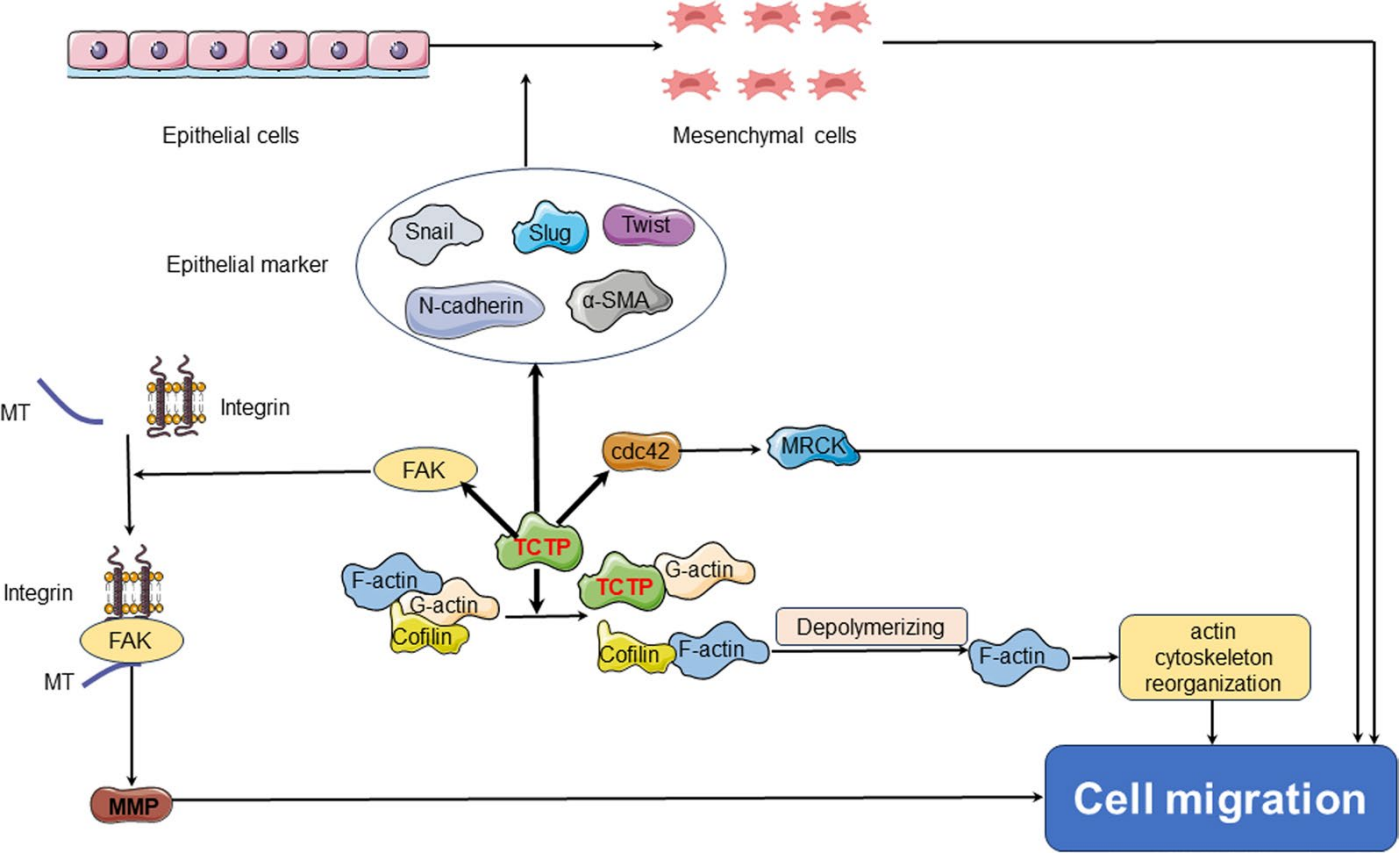
TCTP can be involved in cell migration and tumor invasion. TCTP can upregulate activation of Cdc42 and facilitates cell motility. TCTP can interact with the cell cytoskeleton and release cofilin binding to G-actin by competing with and replacing cofilin. The increase of free cofilin then promotes the binding of the protein to F-actin and exerts its functions. TCTP can promote interactions among FAK, actin cytoskeleton, integrins and extracellular matrix, ultimately facilitating cell migration. TCTP can interact with proteins associated with EMT. *Cdc42* control protein 42 homolog, *FAK* focal adhesion kinase, *EMT* epithelial-mesenchymal transition

Cell migration refers to the cellular process by which cells move in response to migration signals or gradients of specific substances. It involves a coordinated sequence of events, including the extension of cellular protrusions such as lamellipodia or filopodia at the leading edge, establishment of new adhesions, and contraction of the cell body and rear in a temporospatial manner. The cytoskeleton is a network structure composed of protein fibers in eukaryotic cells, which includes microfilaments (MF), microtubules (MT), and intermediate filaments (IF) located within the cytoplasm. These interconnected structures have the ability to polymerize, leading to the facilitation of cell invasion and migration processes. TCTP can enhance cell migration, thus promoting tumor invasion and dropping patient survival rates [113]. Phanthaphol et al. found a close correlation between the upregulation of TCTP and the progression and metastasis of cholangiocarcinoma [114]. Similarly, Jin et al. observed a significant reduction in glioma cell proliferation and invasion after downregulating TCTP expression in the glioma cell line [115]. Currently, TCTP is believed to influence cell migration and tumor invasion primarily through three pathways (Fig. 4).

#### TCTP can regulate cell division control protein 42 homolog (Cdc42) to participate cell migration in cancer

Currently, TCTP is believed to influence cell migration and tumor invasion primarily through three pathways. The first primary way in which TCTP influences cell migration and tumor invasion is through the regulation of the cell cytoskeleton via the small GTPase Cdc42. TCTP exhibits upregulating activation of Cdc42 and facilitates cell motility [116]. In clinical samples obtained from patients with colorectal cancer, a study has revealed a positive correlation between the expression levels of TCTP and Cdc42, as detected by immunohistochemistry [117]. Additionally, Zhang et al. have discovered that the decreased expression of TCTP hinders the invasion and migration of gallbladder cancer cells by reducing the activation of Cdc42 [118]. These findings may provide additional evidence to support the hypothesis that TCTP facilitates cell migration by modulating Cdc42 activity.

#### TCTP can interact with cell cytoskeleton to boost cell migration in cancer

The actin cytoskeleton is a highly dynamic network that regulates cell movement in tumor tissues through the assembly of various structures by the polymerization of globular actin (G-actin) and filamentous actin (F-actin) [119]. TCTP exhibits affinity for both F-actin and G-actin, with a higher affinity for G-actin compared to F-actin. Cofilin can bind to both G-actin and F-actin. The dephosphorylated or active form of cofilin can facilitate cancer metastasis by depolymerizing F-actin, facilitating actin cytoskeletal reorganization, enhancing actin dynamics at the leading edge of the cell, and increasing platelet-based formation and extension [120, 121]. Additionally, molecular structural studies have demonstrated homology between the primary sequence of TCTP and the actin-binding region of cofilin [122]. These aforementioned findings have implied that TCTP could potentially facilitate the dissociation of active cofilin from G-actin. It can be hypothesized that TCTP guides the active form of cofilin towards F-actin, thereby extending the activity cycle of cofilin and ultimately promoting tumor metastasis indirectly. Furthermore, MT reorganization is known to be involved in cellular migration [123]. Ren et al. have revealed overexpressed TCTP in glioblastoma can enhance cell migration by inducing remodeling of the actin cytoskeleton [124]. Apart from the reorganization of MF and MT, certain adhesion molecules, such as focal adhesion kinase (FAK), may play a role in connecting the actin cytoskeleton with integrins, establishing interactions with the extracellular matrix, and ultimately facilitating cell migration. For instance, the signaling pathway of integrin β1-mediated FAK/AKT leads to the expression of matrix metalloproteinase-2 (MMP-2) and MMP-9, which in turn boost the invasion, migration, and adhesion of gastric cancer cells [125]. TCTP can interact with these molecules, triggering tumor invasion and metastasis. Phanthaphol et al. found that knocking down TCTP in cholangiocarcinoma KKU-M055 cells can decline the levels of FAK and pFAK, thereby suppressing cell growth and migration ability [126]. Similarly, in mice with B16F10 melanoma cells, knocking down the expression of TCTP or inhibiting its activity can also suppress the levels of MMP-9, laminin, fibronectin, collagen I and vitronectin [127].

#### TCTP can regulate proteins associated with epithelial-mesenchymal transition (EMT) to promote cell migration in cancer

EMT induces the disruption of intercellular interactions in epithelial cells and acquisition of a mesenchymal phenotype, resulting in increased invasiveness and motility of cancer cells and contributing to tumor metastasis [128, 129]. TCTP can enhance the expression of EMT markers such as N-cadherin, α-smooth muscle actin (α-SMA), and Twist, thereby triggering the activation of MMP-9. MMP-9, in turn, can boost cell invasiveness and accelerate migration by activating the mTORC2/AKT/glycogen synthase kinase 3β (GSK3β)/β-catenin signaling pathway [127]. Xiao et al. have implicated that overexpression of recombinant human TCTP in LoVo cells can increase the expression level of phosphorylated JNK (p-JNK) from the cytoplasm to the nucleus and raise the secretion of MMP-9, ultimately accelerating cell invasion and migration [116]. In contrast, inhibition of TCTP expression can effectively reverse the induction of the EMT phenotype and suppress the occurrence of lung metastasis in melanoma cells [31, 127, 130]. In addition to its role in regulating EMT-related proteins, TCTP also interacts with other tumor proteins implicated in facilitating metastasis. For instance, when the mRNA expression of TCTP is suppressed in LoVo cells, it leads to a concurrent inhibition of the expression and activity of prolactin-3 (PRL-3), a protein known for its involvement in promoting proliferation, invasion, and migration [131, 132]. Moreover, TGF-β1 can be an indispensable regulator of EMT. Mishra DK et al. have revealed that even in the presence of TGF-β1, the invasive and migratory capabilities of cells are diminished upon knockdown of TCTP, indicating the indispensability of TCTP expression, which is regulated by TGF-β1, for driving EMT progression [133].

## Expression and targeted application of TCTP in cancer

### Expression of TCTP in cancer

Multiple clinical studies have provided evidence that TCTP is frequently upregulated in cancerous tissues and is associated with poor prognosis. Its overexpression has been observed in various types of tumors, such as breast cancer, gastric cancer, and cholangiocarcinoma. Amson et al. observed that TCTP exhibits high expression levels in clinical breast cancer samples, while being undetectable in normal breast tissue [27]. The study further highlighted an inverse correlation between the increased expression of TCTP and tumor cell differentiation as well as estrogen receptor levels [27]. Conversely, a positive association was found between TCTP expression and cancer cell proliferation activity. The finding suggested that elevated levels of TCTP expression are indicative of increased tumor invasiveness and poorer clinical prognosis [27]. Simultaneously, a growing body of evidence has suggested that TCTP can hold promise as a clinically relevant biomarker for the identification of low-differentiated and invasive breast cancer [134, 135].

TCTP expression has also been identified in tumors of the digestive system. Helicobacter pylori infection is one of the high-risk factors for gastric cancer. Li et al. applied IHC to detect TCTP expression in gastric cancer and found that it was significantly higher than in adjacent normal tissue [136]. Notably, the expression level of TCTP was observed to be much higher in gastric cancer patients with HP infection than in those without [136], as seen in the pathological sections of their tumor tissues [136]. Further investigation into the mechanism of TCTP involvement in gastric cancer revealed that CDX2 was not expressed in normal gastric mucosal tissue but is highly expressed in gastric cancer [137]. Moreover, recombinant Helicobacter pylori SlyD can induce CDX2 expression and activity in the gastric mucosa in a TCTP-dependent manner, thereby facilitating the development of gastric cancer [136, 138]. Bommer et al. performed TCTP-specific immunohistochemistry on tumor pathological tissues and observed significantly increased expression of TCTP in adenomas, adenocarcinomas, and metastatic adenocarcinomas compared to normal intestinal mucosa or adjacent tissues used as controls [28]. Furthermore, Huang et al. discovered that TCTP played a role in boosting the metastasis of colorectal cancer by modulating the behavior of high mobility group box 1 (HMGB1) and activating the downstream NF-κB signaling pathway [139]. Additionally, TCTP can be involved in the occurrence and prognosis of hepatocellular carcinoma (HCC). Chan et al. conducted quantitative reverse transcription-polymerase chain reaction (RT-qPCR) to detect the mRNA level of TCTP in tumor tissues of HCC patients and found that it was significantly higher than that in adjacent tissues. Moreover, overexpression of TCTP was associated with a significant decrease in the survival rate of patients with HCC [140]. Further investigations into the mechanism of TCTP in HCC revealed that it can trigger mitotic defects and chromosome missegregation during HCC development [140]. Zhang et al. conducted IHC and found elevated levels of TCTP mRNA and protein in invasive and metastatic gallbladder cancer compared to non-invasive and non-metastatic cases [118]. Furthermore, dihydroartemisinin (DHA) was discovered to have the potential to restrain TCTP-dependent metastasis in gallbladder cancer [118].

In addition to its carcinogenic role in breast cancer and digestive system tumors, TCTP has also been implicated in the pathogenesis and progression of lung cancer and reproductive system neoplasms. Kim et al. conducted western blot analysis and observed a significant upregulation of TCTP expression in lung cancer tissues compared to adjacent non-cancerous tissues [141]. Similarly, Sun et al. also observed elevated expression of TCTP in high-grade lung adenocarcinoma tissues compared to low-grade lung adenocarcinoma tissues [92]. Moreover, TCTP was found to play a permissive role in the EMT process of lung cancer by enhancing the expression of vimentin [142]. These findings suggest that TCTP may have potential as a therapeutic target for lung carcinoma. Liu et al. demonstrated that DHA had inhibitory effects on the proliferation of A549 lung cancer cells. Moreover, DHA was found to downregulate the protein expression of TCTP in A549 cells, indicating its potential therapeutic efficacy in the treatment [143].

In addition, previous studies have demonstrated elevated expression of TCTP in malignant prostate cancer and ovarian cancer [126, 144]. Chen et al. further conducted immunohistochemical and Kaplan–Meier analyses on a cohort of ovarian cancer patients, revealing a significant association between high TCTP expression and unfavorable prognosis [145]. Hence, targeting TCTP represents a promising therapeutic approach to significantly enhance the therapeutic efficacy for reproductive system tumors. ASO-mediated knockdown of TCTP, in combination with docetaxel treatment, has emerged as a novel strategy for effectively treating castration-resistant prostate cancer (CRPC) [146]. Simultaneously, sertraline has been discovered to possess therapeutic potential in prostate cancer treatment by simultaneously activating apoptosis and autophagy signaling pathways through deregulation of redox balance [29]. Additionally, previous research has indicated that TCTP is associated with the metastasis of glioblastoma and the occurrence of melanoma, and can serve as a therapeutic target [30, 31].

### Targeted application of TCTP in cancer

As previously mentioned, TCTP demonstrates elevated expression in various types of tumors and is involved in tumor initiation and invasion by modulating cellular proliferation, the cell cycle, cell death, and migration. Therefore, it holds promise as a potential novel therapeutic target for the treatment of malignant cancer in the future**.**

#### Tanshinone IIA

Tanshinone IIA (Tan-IIA), a bioactive compound found in tanshinone, has been demonstrated to possess anticancer effects in certain types of tumors by suppressing TCTP expression. A study has shown that Tan-IIA can downregulate TCTP expression, thereby reducing the expression of the anti-apoptotic proteins Bcl-xL and Mcl-1, while simultaneously increasing the expression of the pro-apoptotic protein Bax [147]. This process can accelerate the apoptosis of malignant cells and inhibit the growth and proliferation of human gastric cancer AGS cells [147].

#### Antihistamines

TCTP is also known as histamine releasing factor (HRF). In this context, antihistamines have been found to have the potential to inhibit the expression of TCTP and may therefore play a role in tumor treatment strategies. It was observed that antihistamine compounds, hydroxyzine, and promethazine, can effectively suppress the expression of TCTP in both breast cancer and leukemia samples, ultimately inhibiting malignant cellular proliferation [112]. Seo et al. have indicated that the antipsychotic drugs levomepromazine and prochlorperazine can inhibit the growth of MCF-7 breast cancer cells by triggering differentiation through binding with TCTP [148]. Levomepromazine and prochlorperazine were observed to induce cell cycle arrest at the G1 phase without triggering apoptosis. Annexin V-PI staining and trypan blue exclusion assay supported the notion that the above-mentioned drugs exerted inhibitory effects on cell growth rather than cytotoxicity [148].

Currently, sertraline, another class of antihistamine medication, has emerged as a focal point in TCTP-related research. Sertraline, initially developed as an antidepressant and primarily utilized in the treatment of depression, belongs to the selective serotonin reuptake inhibitors (SSRIs) category. In 1993, Adam Tramontano and Robert Amson discovered the anticancer properties of sertraline, subsequently opening up new avenues for investigating its potential role in carcinoma [149]. Sertraline can elevate the expression of p53 and decline the expression of TCTP in HCT116 colon cancer cells, thereby disrupting the TCTP-p53 feedback loop and boosting apoptosis [73]. F Ferreira et al. found in a mouse melanoma xenograft model that sertraline is capable of restraining TCTP levels, declining cell viability, suppressing cell migration and colony formation, ultimately inhibiting tumor growth in vivo [31]. In addition, sertraline can reduce the expression of TCTP in breast cancer cell lines, resulting in a significant decrease in cell viability, proliferation, and migration [135]. Apart from its impact on TCTP, sertraline has shown therapeutic potential in treating prostate cancer by suppressing calcium channels [29]. Currently, clinical studies are underway to explore the utilization of sertraline in malignant tumor treatment, such as an ongoing Phase I/II clinical trial involving patients with advanced-stage acute myeloid leukemia (AML) [150]. Hence, further research is needed to fully understand the mechanisms involved and to determine the clinical implications of targeting TCTP with antihistamines for tumor management.

#### DHA

In addition to sertraline, a derivative of artemisinin called DHA has shown promising potential as another tumor-targeting therapeutic agent by specifically binding to TCTP. It has been discovered that DHA can exert antitumor effects in various cellular contexts, including vascular smooth muscle cells, the H1299 cell line, osteosarcoma U2OS cell line, and breast cancer MDA468 and MCF7 cell lines. Mechanistically, DHA can achieve the aforementioned effects by accelerating the ubiquitination and subsequent degradation of TCTP in a protease-dependent manner, triggering reduced expression levels of TCTP [151]. In addition to the aforementioned tumors, DHA has demonstrated efficacy in the treatment of oral cancer by decreasing the levels and activity of TCTP in cancer cells, thereby inhibiting their survival and invasive capabilities [152]. In an A549 lung cancer cell model, DHA has been shown to upregulate the expression of TCTP mRNA while downregulating its protein expression, leading to the suppression of cell proliferation [143]. Moreover, Lucibello et al. have revealed that DHA can specifically target phospho-TCTP and effectively downregulate its expression in breast cancer cell lines, thereby impacting tumor development [153]. In addition to affecting tumor development, DHA has also been found to suppress TCTP-dependent metastasis in gallbladder cancer [118]. Furthermore, the combination of DHA with other anti-cancer drugs has demonstrated promising treatment outcomes. Trastuzumab emtansine (T-DM1) is a targeted therapy for HER2-positive tumors. D'Amico et al. have found that DHA can enhance the sensitivity of HER2-positive breast cancer to T-DM1 by suppressing phosphorylated TCTP levels and disrupting the mitotic process. In their study, the combination of DHA and T-DM1 triggered elevated mitotic abnormalities and microtubule toxicity in the T-DM1-resistant cell lines HCC1954 and HCC1569, leading to enhanced therapeutic efficacy [154].

#### Resveratrol (RES)

RES is a polyphenolic plant compound that can act as a detoxifying agent and elevate the level of the tumor suppressor gene deleted in liver cancer 1 (DLC1) to suppress tumorigenesis [113, 155]. Gao et al. have discovered that the combined treatment of DHA and RES could upregulate the expression of DLC1 while downregulating the expression of TCTP [113]. Additionally, the combination of DHA and RES can regulate the JNK/NF-κB and N-WASP signaling pathways through Cdc42, inhibiting F-actin formation and impeding the migration of HepG2 and MDA-MB-231 cancer cells [113].

#### Other drugs targeted TCTP in cancer

In addition to the aforementioned drugs, oxaliplatin can affect the expression of TCTP, trigger S phase and G2/M phase arrest, promote apoptosis, and suppress proliferation, displaying anticancer effects in colon cancer cells HT29, SW620, and LoVo [156]. Moreover, traditional Chinese medicine ingredients have demonstrated the ability to modulate the expression of TCTP, offering potential for tumor treatment. Chen et al. have discovered that Sann-Joong-Kuey-Jian-Tang can decrease the expression of TCTP in HepG2 cells, thereby exerting suppressing effects on cell proliferation and triggering apoptosis [157]. In HCC J5 cells, curcumin has been found to downregulate TCTP levels, restraining Mcl-1 and Bcl-2 protein expression, and promoting apoptosis through the mitochondrial pathway [158]. Furthermore, curcumin can trigger cell cycle arrest at the G2/M phase by suppressing the expression of Cdc2, thus inhibiting cell proliferation and participating in tumor therapy [158]. RuXian-I has been shown to reduce the upregulation of TCTP, Mcl-1, and Bcl-xL in breast tissue of the mammary gland, which can facilitate apoptosis and potentially inhibit tumor growth [159].

## Conclusion

In general, TCTP plays a crucial role in tumorigenesis and development by regulating cell proliferation, cell death, and cell migration. Furthermore, multiple studies have revealed that TCTP is overexpressed in various types of tumors, including breast cancer, digestive system tumors, lung cancer, reproductive system tumors, and hematologic malignancies, and is associated with poor clinical outcomes. Hence, suppressing TCTP function is a rational therapeutic approach for cancer treatment. Some drugs, such as tanshinone IIA, sertraline, DHA, and RES, have been implicated in targeting TCTP and thus inhibiting tumors at the cellular or animal level. While research on the application of TCTP as a target for cancer management is still in its early stages, it has revealed considerable promise. As further studies delve into the regulation of TCTP expression, its molecular mechanisms, and targeted therapies, this will provide a more comprehensive theoretical basis for the diagnosis and management of carcinoma. Furthermore, targeting TCTP holds the potential to become a new breakthrough in anticancer therapy.

## Data Availability

Not applicable.
